# Perspectives on research needs in healthcare epidemiology and antimicrobial stewardship: what’s on the horizon – Part I

**DOI:** 10.1017/ash.2023.473

**Published:** 2023-11-06

**Authors:** Jonas Marschall, Rachael E. Snyders, Hugo Sax, Jason G. Newland, Thais Guimarães, Jennie H. Kwon

**Affiliations:** 1 Division of Infectious Diseases, Washington University School of Medicine, St. Louis, MO, USA; 2 BJC Healthcare, St. Louis, MO, USA; 3 Bern University Hospital, University of Bern, Bern, Switzerland; 4 Division of Infectious Diseases, Department of Pediatrics, Washington University School of Medicine, St. Louis, MO, USA; 5 Infection Control Department, Hospital das Clínicas, University of São Paulo, São Paulo, Brazil

## Abstract

In this overview, we articulate research needs and opportunities in the field of infection prevention that have been identified from insights gained during operative infection prevention work, our own research in healthcare epidemiology, and from reviewing the literature. The 10 areas of research need are: 1) transmissions and interruptions, 2) personal protective equipment and other safety issues in occupational health, 3) climate change and other crises, 4) device, diagnostic, and antimicrobial stewardship, 5) implementation and de-implementation, 6) health care outside the acute care hospital, 7) low- and middle-income countries, 8) networking with the “neighbors”, 9) novel research methodologies, and 10) the future state of surveillance. An introduction and chapters 1–5 are presented in part I of the article, and chapters 6–10 and the discussion in part II. There are many barriers to advancing the field, such as finding and motivating the future IP workforce including professionals interested in conducting research, a constant confrontation with challenges and crises, the difficulty of performing studies in a complex environment, the relative lack of adequate incentives and funding streams, and how to disseminate and validate the often very local quality improvement projects. Addressing research gaps now (i.e., in the postpandemic phase) will make healthcare systems more resilient when facing future crises.

## Introduction

The 1985 Study on the Efficacy of Nosocomial Infection Control study on the impact of healthcare-associated infection (HAI) surveillance and dedicated infection control measures marks the beginning of modern infection prevention and control (IPC).^1^ During the COVID-19 pandemic, the field has come of age. Many in leadership positions recognized the tremendous value of teams with expertise in infectious diseases, transmission dynamics, surveillance, preventive measures, disinfection, antimicrobial policy-making, and more. It also became clear that the expert networks that IPC teams cultivate are an invaluable resource for healthcare institutions, which otherwise often fall victim to siloing. On the other hand, the pandemic revealed mercilessly how much is still unknown. One example is the intense discussion about what constitutes an infectious aerosol, which most of us believed was well-defined.^2^ Another unresolved question is how to best prevent surgical site infection (SSI), the world’s most common HAI and notable for its multifactorial etiology.

Here, we—a group of IPC professionals with both operational roles and research activities—present an overview of research needs and opportunities, which can serve as a roadmap. Challenges continue to surface as we enter the postpandemic phase of COVID-19. Examples are the global mpox outbreak,^3^ an Ebola virus disease outbreak in Uganda,^4^ the detection of poliovirus in New York City wastewater,^5^ the emergence of *Candida auris* outbreaks around the globe,^6^ and lastly, the reappearance of Respiratory syncytial virus (RSV) and seasonal influenza. As we are getting out of a major healthcare crisis, we should address the identified knowledge gaps and conduct research to advance the field of infection prevention.^7^

## Methods

We conducted a nonsystematic literature review to identify articles that address the needs of research in IPC, offer research agendas, and discuss the future of IPC. We used the search terms “research needs,” “needs assessment,” “knowledge gap,” “road map” in conjunction with reference terms for infection prevention, healthcare epidemiology, antimicrobial stewardship, and others.

In addition, we reviewed statements on research needs by the Society for Healthcare Epidemiology of America (SHEA),^8^ the SHEA-Research Network,^9^ the Association for Professionals in Infection Control and Epidemiology,^10^ the Veterans Health Administration,^11,12^ and the Department for Health and Human Services.^13^ Of note, we found no IPC research agendas published and currently available by the Centers for Diseases Control and Prevention (CDC), the Association for Healthcare Research and Quality, or the European Center for Disease Control.

We identified a set of 10 topics that we believe are relevant for further research (Table 1). Intentionally, we did not single out SARS-CoV-2, first because an excellent SHEA research agenda covers it,^8^ and second because COVID-19 has been a magnifying glass for IPC knowledge gaps in general.

## Chapter 1 – Transmissions and interruptions

COVID-19 transmissions challenged the droplet vs aerosol dichotomy, which is likely to be replaced by an understanding of a *continuum* of particle size.^2^ Other transmission factors should be studied in-depth, such as the means of particle emission (speaking, coughing), setting, humidity, ventilation, air suspension, mode of ingestion and modulations thereof such as masks, and, lastly, the pathogen and its infectious dose. Proximity and exposure time are probably the most relevant determinants,^14^ but the survival time of live virus in particles needs to be elucidated further.^15^ We anticipate additional insights from collaborative work between IPC experts, physicists, and ventilation engineers. Hospitals in warmer climate zones that rely heavily on air conditioning should have AC systems reviewed as part of their IPC strategy.^16^ The concept of aerosol-generating procedures helped direct N95 masks to situations considered high risk^17^ but is based on incorrect assumptions: it needs to be replaced with a better model anchored in additional aerosol emission studies.

A good part of reservoirs and transmission routes remain unexplained. Potential environmental sources may be devices, textiles, room and furniture surfaces, sanitary installations, and hospital food (which collectively may harbor the “hospital microbiome”); we need to characterize these reservoirs and risks and then address them.^18^ For inanimate surfaces, UV-C emitting devices are among the best-studied measures to decrease surface contamination,^19,20^ yet they are costly, require specific training, cause room downtime, and do not affect all microbes equally. This and other cleaning modalities, the disinfectants, the evaluation of cleaning thoroughness, and the optimal training of environmental service workers are topics of further research.^21^ Emerging pathogens deserve special attention regarding suitable disinfectants.^22^

We have an incomplete understanding of what measures can best break transmission chains. We experienced a large vancomycin-resistant *Enterococcus* outbreak^23^ and implemented a multitude of measures^24^; however, without a sequential approach one cannot tell which measure was most or least helpful. Although the “bundled approach” has been popular at least since Pronovost’s 5-item bundle for Central line-associated bloodstream infection (CLABSI) prevention,^25^ we believe the deconstruction of bundles to determine the most efficient components should be among the next goals in IPC.

On a patient level, decolonization regimens were conceived and tested for various multidrug-resistant organisms (MDROs); however, only methicillin-resistant *Staphylococcus aureus* decolonization and preoperative *S. aureus* decolonization have become mainstream. In decolonization trials for extended-spectrum betalactamases and carbapenemase producers, no long-standing success was seen.^26^ There should be explorations into additional strategies to shorten MDRO colonization. While not technically decolonization but rather modulation, microbial interference^27^ and microbiome substitution to counter dysbiosis^28^ are likely to become attractive intervention avenues.

To date, screening strategies after exposures or during outbreaks have been based on geographical proximity (i.e., sharing the same room) and not on shared devices or “shared” healthcare personnel (HCP). Smarter screening approaches should be evaluated that take the entire transmission network into consideration.

Lastly, ardent debates about the duration of isolation are not infrequent. The fact that we have greater control over later stages than the beginning of contagiousness obviously invites us to delineate the natural course of an illness. However, many recommendations aim for maximum risk reduction, when in fact we need to study the balance between what is acceptable to patient and provider and what serves for a reasonable risk reduction. Public health authorities should adopt this approach, too.

**Table 1.** Research needs and opportunities in infection prevention and control (IPC)

Chapter	Title	Addressed topics
1	Transmissions and interruptions	Transmission concepts, interventions to interrupt the transmission chain, environmental cleaning, decolonization
2	PPE and other safety issues of occupational health	Protective equipment, vaccination, hand hygiene
3	Climate change and other crises	Effect of climate on SSI, antimicrobial resistance (AMR), zoonoses, emerging infections, human encroachment into remaining biotopes, how to strengthen healthcare for crises, sustainability
4	Device, diagnostic, and antimicrobial stewardship	Stewardship forms, device reprocessing
5	Implementation and de-implementation	Behavioral change, learning and unlearning, discontinuing low evidence measures
6	Healthcare outside the acute care hospital	Outpatient care, nursing homes, long-term care facilities
7	Low- and middle-income countries	Inequality and scarcity, low cost interventions, knowledge transfer, travel screening
8	Networking with the neighbors	Collaborations with related fields, such as patient safety and quality, microbiology and data science; explore the interfaces between areas
9	Novel research methodologies	Emulated trials, cluster-randomized trials (CRT), machine learning, design research networks, artificial intelligence
10	The future state of surveillance	Early detection, automated surveillance, optimizing the feedback loop

Note. The research needs are presented in topical groups with overarching labels; notably, there is overlap between many of these groups.

## Chapter 2 – Personal protective equipment and other safety issues in occupational health

The personal protective equipment (PPE) follows the prevailing transmission concept. Choosing the optimal PPE does not just derive from scientific evidence, though, but it is influenced by the economic environment, supply chains, its usability design, and how swiftly PPE utilization can be taught. The latter opens up an entire field of research, the behavioral science of infection prevention.^29^ Notably, to our knowledge, most institutions do not provide specific training for proper PPE donning and doffing procedures outside of the use of N95 respirators, which occurs in the form of “fit testing.”

Default gowning and gloving for contact precautions are meant to make things easy; however, not every patient interaction requires the use of a gown (it may only be necessary when in immediate physical contact with the patient). Research that explores situation-dependent approaches is needed and will appeal to HCP for the nuanced perspective on the clinical interaction, even if demanding a greater cognitive effort. Moreover, it has the potential to reduce expenditure and hospital waste and drive other behavior in a beneficial way, for example, hand hygiene adherence.^30^

We often intend to eliminate any exposure risk in its entirety; it is intuitive to want to protect the employee’s eyes from splashes of a patient’s respiratory secretions. However, a proper risk assessment would determine how often these splashes occur, if secretions reach the employee’s conjunctivae, whether employees have subsequently developed a (respiratory) virus infection, and how severe this was. Based on this, a well-balanced decision should result as to in which situations eye protection is recommended. In addition, there should be more comparative research into different measures of protection. For example, a major question surrounding COVID-19 prevention, whether to wear a medical mask or an N95 respirator, was only addressed in one randomized trial so far.^31^

Hand hygiene is a key element of hygienic behavior among the workforce; however, this is not a perfect success story. The evidence base is relatively weak.^32^ The workforce’s poor hand hygiene adherence is noteworthy (40% in one systematic review^33^) and this is unlikely to be much better in times of staff shortage and rising case complexity. How to address those individuals with consistently poor adherence is unresolved. Moreover, the standard of observing adherence by trained HCP in the patient room is incredibly time intensive and fraught with bias. This needs to be replaced with smarter ways of measuring adherence. Some ideas involve electronic barriers or other alerts if the patient is approached without prior hand hygiene, while others have looked into using surrogate markers such as alcohol-based hand rub consumption.^34,35^ Novel interventions that do not require large up-front investments are much needed.

For occupational health, another question is how to motivate employees to obtain immunization for protection. Some healthcare systems have moved to mandatory flu vaccination,^36^ which is straightforward but can create legal problems and requires much logistical effort. Most systems probably continue using a carrot and stick approach, with mixed results.^37^ As for SARS-CoV-2, many healthcare systems around the world have opted for making the primary vaccination series a requirement, and we are still to see the effect of these pandemic-era policies on future influenza vaccination uptake. The debate about what should be expected from the individual for the greater good of a population’s health is not over.^38^ Future research should look into incentivizing HCP for vaccination in novel ways.

Lastly, educating the entire healthcare workforce in IPC should be a core topic for patient safety and quality but varies greatly in how it is conveyed and sometimes is missing entirely. There should be novel routes of education, as in the form of IPC webinars for interested HCP, virtual sessions the attendance of which can be made a requirement for clinical work, simulation-based learning, and other forms of qualification in IPC. We specifically recommend striving for the right balance between expecting simple policy adherence and educating HCP on the infection risks in our complex healthcare environment.

## Chapter 3 – Climate change and other crises

Climate effects and climate change on a global scale have only recently been addressed by IPC research, mostly by studying the seasonality of SSI where rates were higher in warmer periods of the year.^39,40^ It remains to be determined why seasonality matters when the actual procedure occurs in a temperature-regulated operating room. Also, it is unclear whether this should translate into climate-sensitive scheduling of procedures or other measures. The association with climate zones needs investigation for all types of HAIs, including variations in predominant microbial species and resistance patterns.^41^

Antimicrobial resistance is likely to increase with climate change.^42^ Multiple aspects deserve attention in this line of research. For example, what is the prevalence of MDRO in sanitary and wastewater systems in function of the environmental temperature? How do typical CLABSI and CAUTI pathogens change as the average temperature increases? What is the effect of migrant movements from areas that are becoming inhabitable on the populations receiving them, in terms of MDRO shifts? What about global travel as a vector? What increases in AMR can be projected, depending on how much a given region is affected by climate change?

It is likely that future challenges develop at the intersection of human encroachment with natural reservoirs and will hit populations that initially lack immune protection. An example is the recent Ebola outbreaks in West Africa, where the animal host (or an intermediate animal) transmits the disease. Zoonotic infection research and working with veterinarians to better understand risks originating from animal sources are essential—this should be part of pandemic preparedness on a global level.^43^

Given the wide-ranging effects of the pandemic, we also think that a more holistic and nuanced approach to preparedness is warranted. Prior to the COVID-19 pandemic, healthcare systems around the world have written or updated pandemic plans for *influenza*, ignoring the fact that the most challenging viruses of recent times were coronaviruses. Given that pandemics usually occur in intervals of decades, pandemic planning was not always given enough attention (and hence, the level of preparedness of institutions varied greatly). Pandemic preparedness can be improved by anticipating pandemics with different pathogens, playing through different scenarios, conceptualizing incident command centers to be activated when needed, and establishing more resilient equipment and medication supply chains. Moreover, if they have not done this already, we strongly believe healthcare systems should conduct debriefings and learn as much as possible from their pandemic experience. The goal should be lean, efficient, thought-through processes that can be readily deployed.

Healthcare systems need to also have outbreak management plans in place that help them jump into action when the first signals of an unusual pathogen appear or detection of a specific pathogen increases. There are many resources available, including toolkits from the CDC (https://www.cdc.gov/hai/outbreaks/outbreaktoolkit.html). Research should look into how to provide a standard on a national level and how to empower healthcare systems to have such plans ready for use.

Lastly, we wanted to mention sustainability, environmental aspects, and (hospital) waste. Although not traditional IPC topics, they present major societal challenges and should be considered in future research as they tie into infection prevention. One example is endoscopy where single use or partially disposable devices are beginning to be marketed^44^ and create significant waste, all in the name of avoiding risk to the patient that could come from faulty reprocessing of multi-use devices.

## Chapter 4 – Device, diagnostic, and antimicrobial stewardship

Device use correlates with infection risk and has therefore been the target of efforts to prevent the device-associated infections CAUTI, CLABSI, and ventilator-associated pneumonia.^45^ Studies should elucidate what novel surfaces or coatings may prevent colonization and infection (given that there is reasonable evidence for coated vascular catheters but no convincing data to implement coated urinary catheters or endotracheal tubes), how to reduce catheter use and shorten the duration of catheterization, and, specifically, the comparative risk of different forms of vascular access should be investigated. For urine drainage, there are noninvasive options, and the proportion of infections avoided by such alternatives should be determined. A very first, but extremely important step is to find ways to ensure that documentation of device use is accurate.

Another device-related area of research need is the high-level disinfection (HLD) of reusable devices such as endoscopes, the process of which is quite complex (with multiple opportunities for contamination). We think there is a huge need for standardized education on HLD, in particular outside of the relatively well-regulated central sterilization departments, for example, in otorhinolaryngology and gastroenterology clinics, and research into the best delivery of such education. We consider augmented reality a particularly exciting way of enhancing training that merits further study. Also, quality indicators of the disinfection process need to be evaluated,^46^ as process metrics in HLD are still in their infancy.

There are further types of stewardship, which often lie in the hands of IPC experts, such as *diagnostic* stewardship. A thorough diagnostic work-up is desirable in view of downstream antibiotic management. Not all diagnostic testing is appropriate or even necessary, though. In fact, the proportion of unnecessary tests appears to be at a staggering 40%–60%,^47^ and these test results may lead to unnecessary antibiotic administration, unnecessary additional testing, or even unnecessary procedures. Therefore, research into the optimal utilization of tests (including blood cultures and respiratory panels, among others) is needed. This may include restricting certain testing options, avoiding duplicate testing, devising order sets with decision-support elements, exploring rapid test modalities, optimizing the feedback loop to educate, and present test results so as to nudge the provider to “do the right thing.”^48^

From diagnostic stewardship, there is a direct line to antimicrobial stewardship,^49,50^ the art and science of optimizing antimicrobial use in clinical practice, which has matured into its own field over the past 20 years. We believe there are numerous aspects that should be studied further.^51^ These include smart feedback and education to providers regarding their antimicrobial use, implementing barriers to (over-)using antibiotics of last resort, finding ways to reduce overlong administration such as for peri-procedural prophylaxis, and displaying the data for tracking purposes.^52^ Much about this centers around educating providers that “more of an antimicrobial” in their eyes will not always translate into a benefit for the patient or the public health. Determining the adequate staffing level of pharmacists, clinicians, and other healthcare professions for antimicrobial stewardship, a younger and less established field than IPC, is a common challenge and requires crafting a robust business case and more data regarding best practice.

## Chapter 5 – Implementation and de-implementation

Not infrequently, IPC experts will have a good idea of what measures should be taken to decrease infection rates. However, how to educate and incentivize and eventually get the workforce to do those things is a science in itself, and it is called dissemination and implementation (short, D&I). D&I has begun to appear in IPC research in recent years.^53,54^ It may provide an explanation for why a specific intervention does not seem to work (when in fact, it just was not implemented well).

A large part of turning IPC policies into reality depends on soft skills of experts. How to convince, how to steer behavior, and how to negotiate change are all common questions but they are not usually taught well. Future research should engage behavioral science to understand how the IPC expert can be an “influencer” and modify healthcare worker behavior.^29,55^ Simulation and hands-on learning should be studied more extensively as should novel ways of delivering education content (e.g., the “room of horrors” approach to identifying medical errors in a mock patient room^56^). Moreover, the concept of “change management” has been used in the pharmaceutical industry for decades but has become more commonly heard in healthcare only recently; it deserves being applied to IPC endeavors, too.^57^

*De-implementation* is the process of identifying and systematically ending health interventions that are low value (e.g., ineffective) and overused (e.g., wasteful) to maximize available resources and improve patient care. A current example of much-needed de-implementation is contact precautions in COVID-19; although a negligible proportion of COVID-19 infections are acquired via direct contact, the CDC still recommends contact precautions. This is a suitable target for de-implementation and would free up time and resources that can then be used otherwise. In a similar vein, unnecessary “habits” in medical care may need to be “un-learned”. One recent example is a high-quality trial being conducted to reduce postoperative antibiotic duration in children.^58^ Targets for de-implementation include PPE components, the use of insertable devices, antibiotic administration, diagnostic work-up, and other measures.
