# Perspectives on research needs in healthcare epidemiology, infection prevention, and antimicrobial stewardship: what’s on the horizon—Part I – CORRIGENDUM

**DOI:** 10.1017/ash.2023.513

**Published:** 2023-11-30

**Authors:** Jonas Marschall, Rachael E. Snyders, Hugo Sax, Jason G. Newland, Thais Guimarães, Jennie H. Kwon

**Affiliations:** 1 Division of Infectious Diseases, Washington University School of Medicine, St. Louis, MO, USA; 2 BJC Healthcare, St. Louis, MO, USA; 3 Bern University Hospital, University of Bern, Bern, Switzerland; 4 Division of Infectious Diseases, Department of Pediatrics, Washington University School of Medicine, St. Louis, MO, USA; 5 Infection Control Department, Hospital das Clínicas, University of São Paulo, São Paulo, Brazil

In the initial publication of this article^1^, the phrase “infection prevention” was omitted from the title.

The article title has been updated to include this phrase.
